# Morphological and micro-tomographic study on evolution of struvite in synthetic urine infected with bacteria and investigation of its pathological biomineralization

**DOI:** 10.1371/journal.pone.0202306

**Published:** 2018-08-14

**Authors:** Muhammed A. P. Manzoor, Balwant Singh, Ashish K. Agrawal, Ananthapadmanabha Bhagwath Arun, M. Mujeeburahiman, Punchappady-Devasya Rekha

**Affiliations:** 1 Yenepoya Research Centre, Yenepoya (Deemed to be University), Mangalore, Karnataka, India; 2 Department of Urology, Yenepoya Medical College, Yenepoya (Deemed to be University), Mangalore, Karnataka, India; 3 Technical Physics Division, Bhabha Atomic Research Centre, Indore-Mumbai, India; VIT University, INDIA

## Abstract

Pathological biomineralization in the urinary system leads to urolithiasis. Formation of kidney stones involves a series of events during which they undergo morphological and mineralogical changes. We investigated the mineralization of biogenic struvite (*in vitro*) and examined the transformation of distinct interior and exterior structure of struvite. *In vitro* crystallization of struvite was performed in the presence of two bacteria that were originally isolated from the kidney stone patients. Morphological evaluation was carried out using SR-μCT as well as FESEM, XRD and FT-IR. Characteristic internal 3-D morphology and porosity of the stones were studied. For comparison, patient derived struvite stones were used. From the results obtained, we report that the presence of bacteria enhances the crystallization process of struvite *in vitro*. A series of time-resolved experiments revealed that struvite crystals experienced a significant morphologic evolution from pin pointed structure to X-shaped and tabular morphologies. These X-shaped and unusual tabular habits of struvite resembled biogenic morphologies of struvite. SR-μCT showed similarities between the patient derived and the *in vitro* derived struvite crystals. In conclusion, these experiments revealed that the bacteria play a major role in the specific morphogenesis of struvite and can able to control the nucleation, modulate crystalline phases, and shape of the growing crystal.

## Introduction

Urolithiasis (kidney stones) developed as a result of urinary tract infections makes up approximately 10–15% of all stones worldwide [1]. Struvite is the most frequent type of infection stone with mineral composition of magnesium ammonium phosphate hexahydrate (MgNH_4_PO_4_·6H_2_O). It is associated with infection caused by urease-producing microorganisms [2–4]. Breakdown of urea of urine by bacterial urease into carbon dioxide and ammonia leads to alkaline pH and elevates the concentration of NH_4_^+^, PO_4_^3-^ and CO_3_^2-^ [5]. These ions present in urine subsequently combine with Mg^2+^ to precipitate into struvite [6]. Among all stone types, struvite can proliferate and occupy the renal calyces and pelvis; significantly damaging the epithelium of the internal renal walls [1, 7]. Struvite is one of the most common aetiologies of staghorn morphology and if untreated it can result in complications like pyonephrosis, perinephric abscess formation, or xanthogranulomatous pyelonephritis with the eventual loss of the kidney function [7–9].

Crystallization and morphogenesis of struvite have been investigated by different researchers [6, 7, 10, 11]. Morphological variations of struvite crystals may depend on the factors such as mineral super saturation, growth kinetics, pH, specific gravity and by the presence of modulators or inhibitors. Biogenic struvite can be grown from small subunits and it can develop into well-defined faces with specific structures [7, 12]. Structure, morphology and physical properties of the stone influence greatly on the diagnosis and treatment planning.

The presence of bacterial imprints in the kidney stones have always been a subject of investigation, however very few studies have reported the presence of bacterial imprints in kidney stones with small and large nanocrystals, such as carbonated apatite and struvite respectively [13]. Among the various analytical tools μCT is employed to get information on the texture, microstructure, and mineralogical details of kidney stones, as it permits the cross-sections across the samples [14]. We applied SR-μCT along with other techniques to evaluate the growth, morphology, and habit of struvite formed in the presence and absence of two urease producing bacteria isolated from infectious kidney stones. These bacteria were chosen to mimic the biogenic crystallization and growth of struvite by single diffusion gel technique in synthetic urine. The details obtained from the *in vitro* generated stones were compared with patient derived struvite stones.

## Materials and methods

### Ethics statement

All procedures performed in studies involving human participants were in accordance with the ethical standards of the institutional (Ethical clearance approval number YUEC.022/16), national research committee and with the 1964 Helsinki declaration and its later amendments or comparable ethical standards. The study was approved by the Institutional Ethics Committee (IEC) and Scientific Review Board (SRB) of Yenepoya (Deemed to be University), India. Written informed consents were obtained from each participating patient prior to specimen collection.

### Chemicals and media for *in vitro* crystallization experiments

All chemicals used were purchased from commercial sources. Sodium metasilicate (Na_2_SiO_3_), ammonium dihydrogenphosphate (NH_4_H_2_PO_4_) and magnesium acetate solution (C_4_H_6_MgO_4_) were of analytical grade. These chemicals were dissolved in Milli-Q Type 1 water. The synthetic urine used for biogenic crystallization was prepared according to Griffith et al. [15]: CaCl_2_. 2H_2_O, 0.651; MgCl_2_. 6H_2_O, 0.651; NaCl, 4.6; Na_2_SO_4_, 2.3; KH_2_PO_4_, 2.8; KCl, 1.6; NH_4_Cl, 1.0; sodium citrate, 0.65; sodium oxalate, 0.02; urea, 25.0; creatine, 1.1; and tryptic soy broth, 10.0 (g/L). The content of the mineral components in synthetic urine corresponds to mean concentration found in 24 h period in normal human urine.

### Patient-derived stones and bacterial experiments

Surgically removed struvite stones were collected from patients who had undergone percutaneous nephrolithotomy (PCNL). Presence of struvite composition was confirmed by Fourier transform infrared spectroscopy (FT-IR) and X-ray powder diffraction (XRD). The collected stones were washed with sterile distilled water, homogenized and plated onto MacConkey agar, and Nutrient agar (Hi-media, Mumbai). The plates were incubated aerobically at 37°C for 24 h. Growth of bacteria was observed and total numbers of colonies were counted.

### Bacterial culture, DNA isolation and 16S rRNA gene sequencing

For bacteriology, kidney stone homogenates were transferred to sterile distilled water and further inoculated to MacConkey agar and Nutrient agar and incubated for 24 h at 37°C. Following incubation, each colony was counted using a digital colony counter and further processed for Gram staining. The bacteria were taxonomically identified using 16S rRNA gene sequencing. Briefly, genomic DNA was extracted from the bacterial cells harvested from tryptic soy broth by phenol: chloroform extraction method. The concentration of resulting DNA was measured using Nanodrop-1000 (Thermo Scientific, USA). The genomic DNA was amplified in polymerase chain reaction using standard primers namely 27F (5′-CCAGAGTTTGATCMTGGCTCAG-3′) and 1490R (5′-GGTTACCTTGTTACGACTT-3′) [16]. Amplifications were performed in a Thermal Cycler under the conditions: initial denaturation at 94°C for 5 min, followed by 30 cycles of denaturation at 94°C for 1 min, primer annealing for 1 min at 55°C, and primer extension for 1 min at 72°C with a final extension period at 72°C for 10 min. The amplified product was purified by precipitation using 20% Polyethylene glycol (using 2.5 M NaCl) and sequenced both using forward and reverse primers using the BigDye Terminator v3.1 Cycle Sequencing Kit and ABI 3730 XL DNA Analyzer (Applied Biosystems Inc, USA).

### Crystallization experiments

Initial early stage crystallization was carried out directly and observed using light microscopy. Single diffusion gel growth technique was used to grow the struvite crystals as described elsewhere [3, 10, 17]. Briefly, sodium metasilicate solution of specific gravity 1.05 was used to prepare the gel. An aqueous solution of ammonium di-hydrogen phosphate (0.5 M) was mixed with sodium metasilicate solution in an appropriate amount to set the pH of the gel at 7.0. Bacteria cultured in trypticase soy broth overnight at 37°C were suspended in synthetic urine to a concentration of 5x10^5^ CFU/mL. Prior to gelation experiments, bacterial growth in the synthetic urine was assessed to check the viability. After the gelation took place, 20 mL supernatant solutions of 0.5 M magnesium acetate was gently poured on the gels in the test tubes (140 mm length and 25 mm diameter) without disturbing the gel. The same compositions were also used for control without bacteria in synthetic urine. All experiments were performed in triplicate under aseptic conditions incubated at a temperature of 37 ± 0.5°C. During the experiment, pH of the samples was measured using a digital pH meter and samples from different stages of the crystallization process were harvested for investigations. Structural attributes of the *in vitro* generated struvite crystals and patient-derived struvite were studied by FT-IR, XRD, and FESEM as explained elsewhere [18].

### SR- μCT imaging and 3D reconstruction

**Fig 1:**
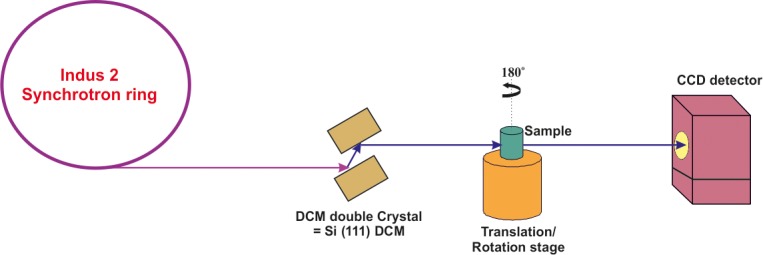
Schematic diagram of imaging beam line, showing beam propagation path and configuration of various components.

Struvite crystals grown in the presence and absence of bacteria were studied by X-ray micro-tomography experiments conducted at X-ray Imaging Beamline (BL-04) on Indus-2 Synchrotron Source RRCAT, Indore (Fig 1) [19]. The 2D projection images for all the samples were acquired at 15 keV beam energy obtained from fixed exist Si (111) double crystal monochromator (DCM) at 150 mm sample to detector distance. The projection images were acquired with rotation step size of 0.2^o^ for total 180^o^. The exposure time taken was 500 m sec. for each projection including the bright and dark field images. High-resolution CCD detector with pixel size 4.5 and an active area of 4008 x 2670 pixels was used to acquire the experimental images. The data acquired was preprocessed, reconstructed and 3D volume rendering was done by Drishti visualization software [20]. Quantitative analysis of the SR-μCT data on porosity variations was carried out using ImageJ software. For calculation of porosity, the 3D volume was first binarized using Otsu method of threshold followed by noise removal using the median filter. Patient derived stones were also similarly investigated.

## Results

### Bacterial isolates and taxonomic identification

Two urease positive strains namely, YU22S and YU27S isolated from the stone culture of the patients with struvite stones were used for *in vitro* struvite crystallization. Based on 16S rRNA gene sequencing, the isolates YU22S and YU27S were identified respectively as *P*. *aeruginosa* and *E*. *cloacae*. The NCBI GenBank accession numbers for the sequences are MH021663 and MH021673 respectively. The cultures were deposited at Microbial Culture Collection Center (NCMR, Pune) with culture collection numbers; YU22S as MCC 3101 and YU27S as MCC 3111.

### *In vitro* struvite growth in the presence of urease producing bacteria

**Fig 2:**
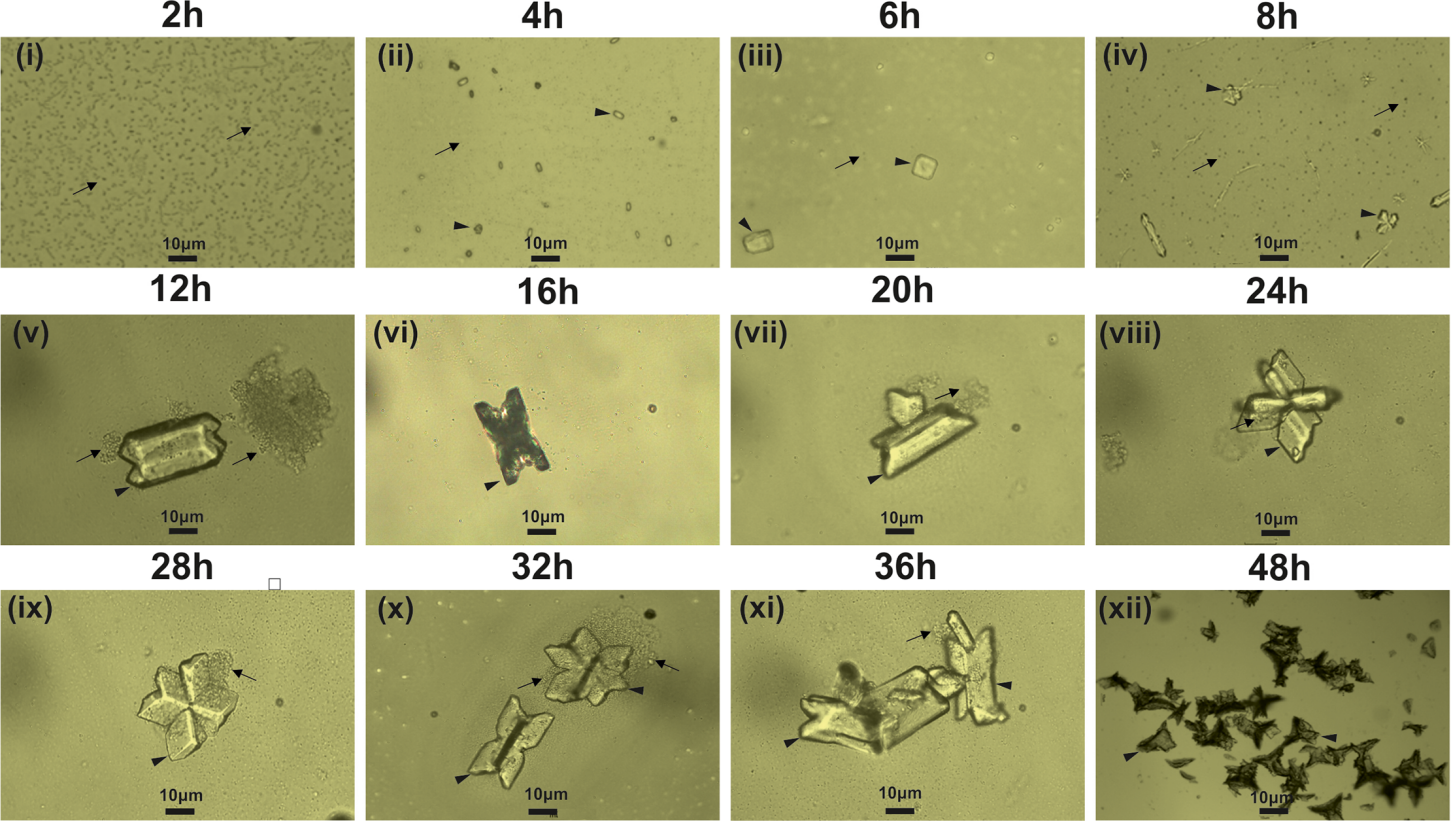
Temporal growth pattern of struvite crystals grown in artificial urine infected with bacteria (2–48 h). Arrowhead shows crystals and arrow shows bacteria.

The early stage (10 min- 48 h) crystallization of struvite revealed by the initial experiment is given in Fig 2. During this, the size varied from a few microns to more than 10 microns. Struvite crystals in both control and bacteria induced sets exhibited a coffin-like habit with well-defined planes growing to X-shaped dendrite crystal (Fig 2iv–2vi). The bacteria induced struvite subsequently formed twins (Fig 2viii–2xi).

### Effect of bacteria on the morphology and aggregation of struvite crystals

The time-resolved experiments revealed the growth of small dendritic type struvite crystals immediately after the addition of supernatant solution, at the gel-liquid interface. The initially grown pinpoint crystal tubes tend to nucleate randomly throughout the test tube and on the gel surface (S1 Fig). In the presence of bacteria, the pH of the supernatant solution at this stage increased gradually.

At the end of third-day, the crystals grown in all the conditions appeared as single crystals exhibiting coffin-like habit with well-defined planes. In the presence of *P*. *aeruginosa* and *E*. *cloacae* X-shaped dendrite crystals were observed the end of the fifth day, with high accumulation of crystals compared to control. The aggregation of crystals was rapid in the presence of bacteria compared to the control sets. At pH greater than 9, the hopper like crystals were also observed coexisting with dendrite-like crystals.

### SR- μCT of struvite stone

**Fig 3:**
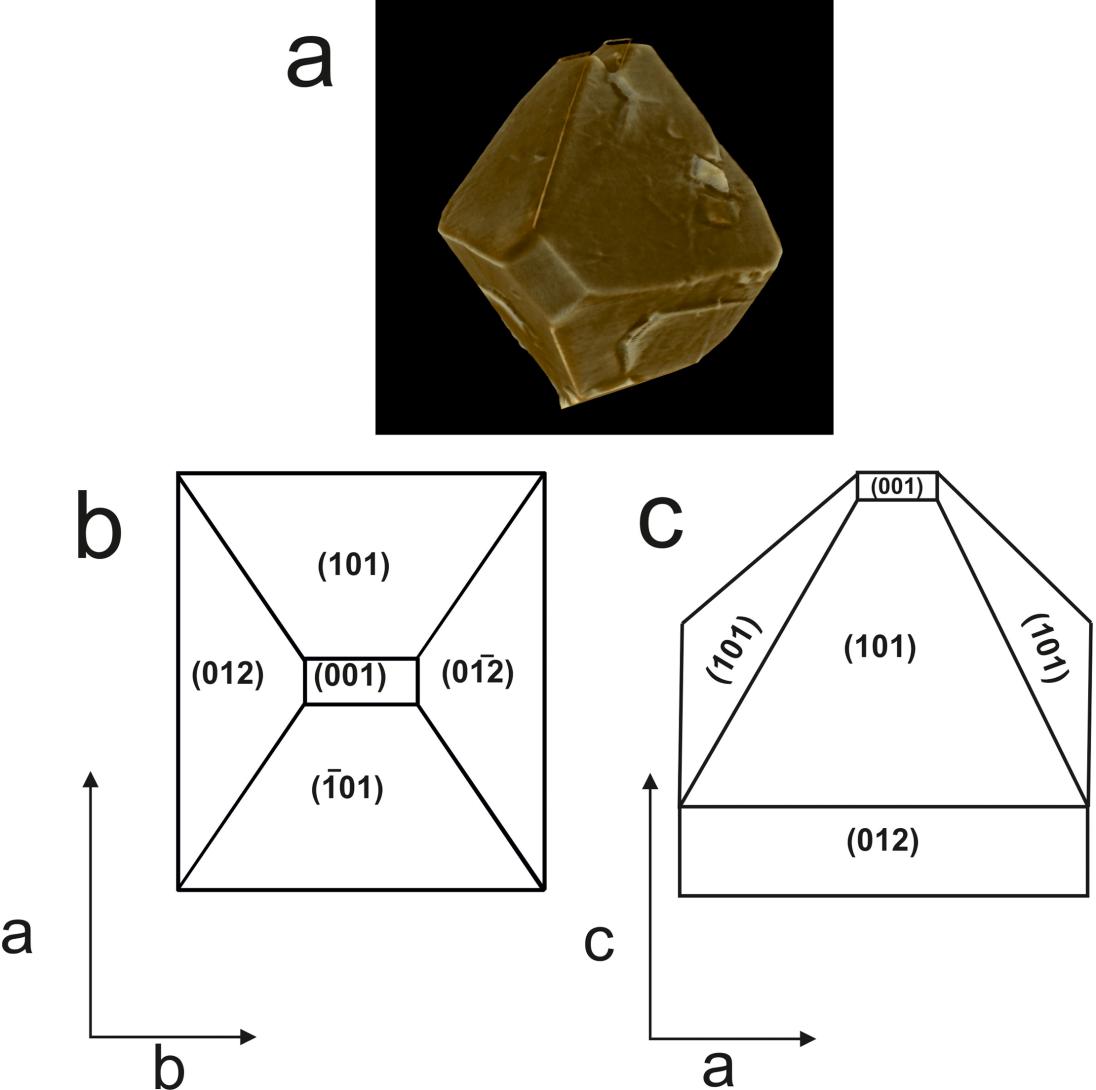
(a) SR-μCT 3-D images showing the early stage of struvite crystallization (b, c) schematic representation of struvite morphology from artificial urine in the presence of urease positive bacteria.

**Fig 4:**
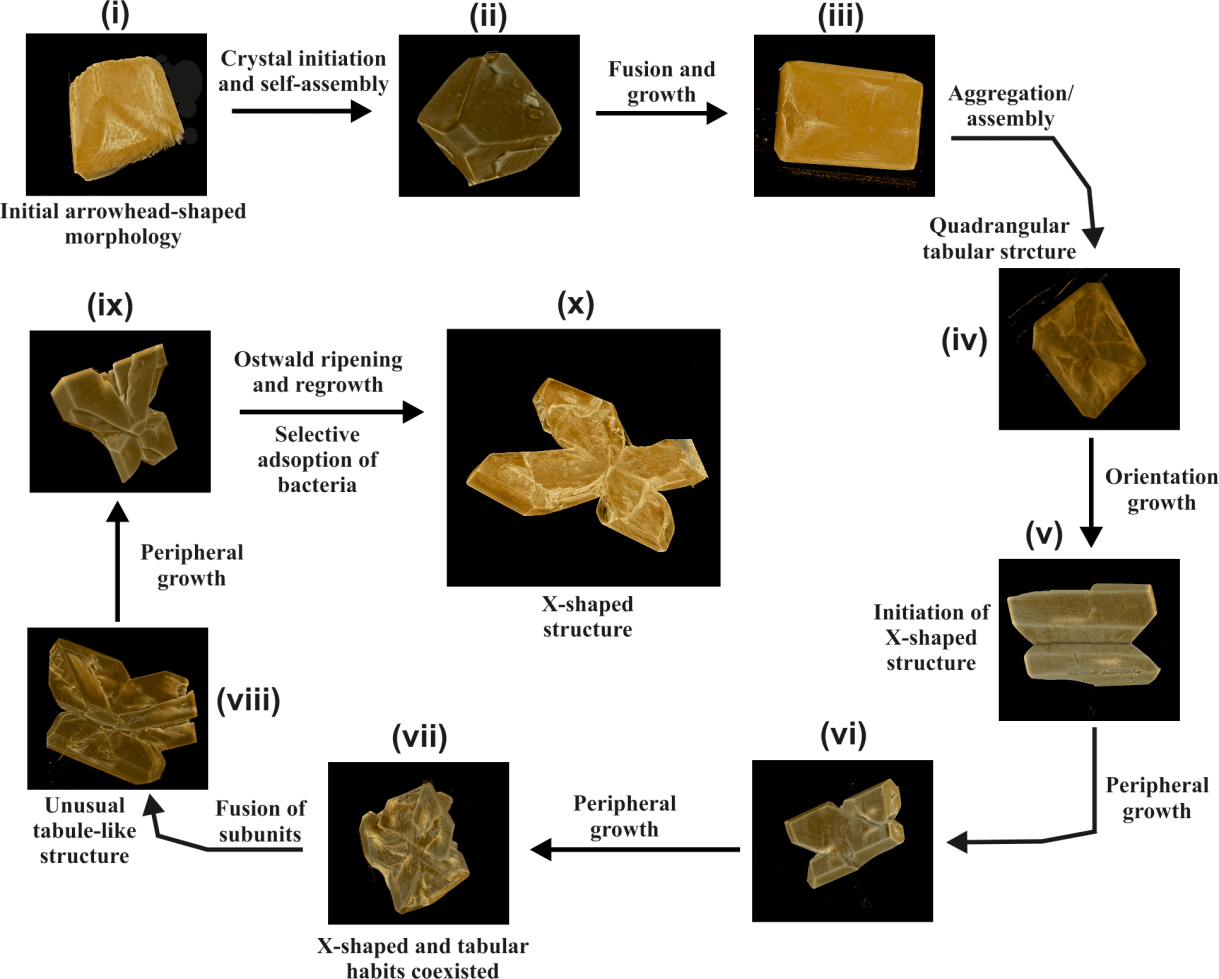
SR-μCT 3-D images showing the morphological evolution process of struvite in the presence of bacteria.

At the initial stages of struvite mineralization, crystals displayed well-defined faces (Fig 3). The arrowhead-shaped crystals grown in the presence of bacteria showed preferential growth in (001), (101), (Ῑ01) and (012) faces (Fig 3). The SR- μCT of struvite crystals is presented in Fig 4. At the second stage, morphology of the crystals formed in the bacterial set was distinct presenting an intermediate form between the single and dendrite-like crystals (Fig 4iii). Distinct X-shaped structures developed further in the presence of bacteria (Fig 4iv). Growth of these X-shaped crystals occurred along the (111) direction of every assembled subunit. The crystals formed in the control also showed the X- shaped morphology and the general morphology of the X- shaped crystals displayed differences between the control and the bacterial induced crystallization. The patient derived stones presented layered pattern and exhibited void spaces with cracks inwards from the outer surfaces (S2 Fig).

The percentage porosity showed a distinct variation at different stages of struvite formation in the presence and absence of bacteria (S1 Table). The mean porosity of bacterially induced struvite crystal increased from initial 0.0098% to final stage 11.45%. But, in the control the porosity decreased from 12.17% to 8.43% from initial to final stage.

### FESEM

**Fig 5:**
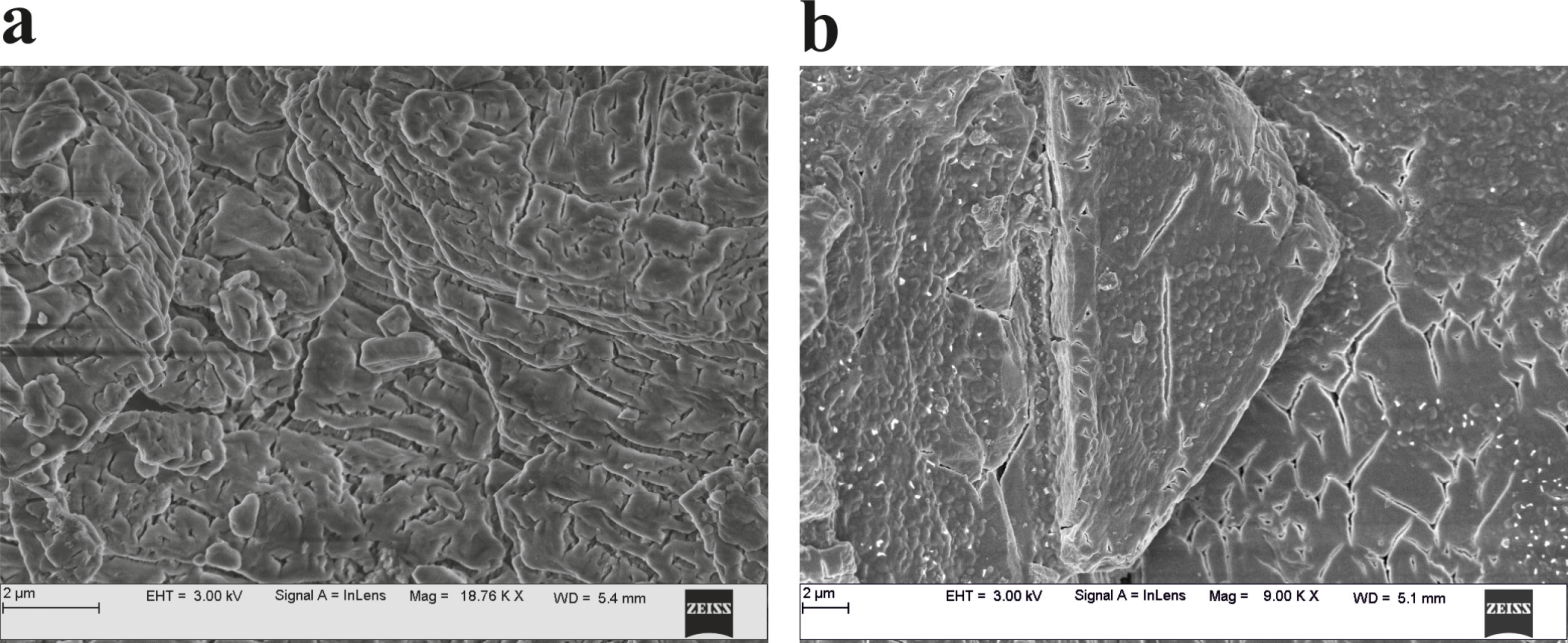
Representative FESEM micrographs of struvite crystals grown in artificial urine revealing the porous structure and mesoscopic arrangement (a) control (b) in presence of bacteria.

**Fig 6:**
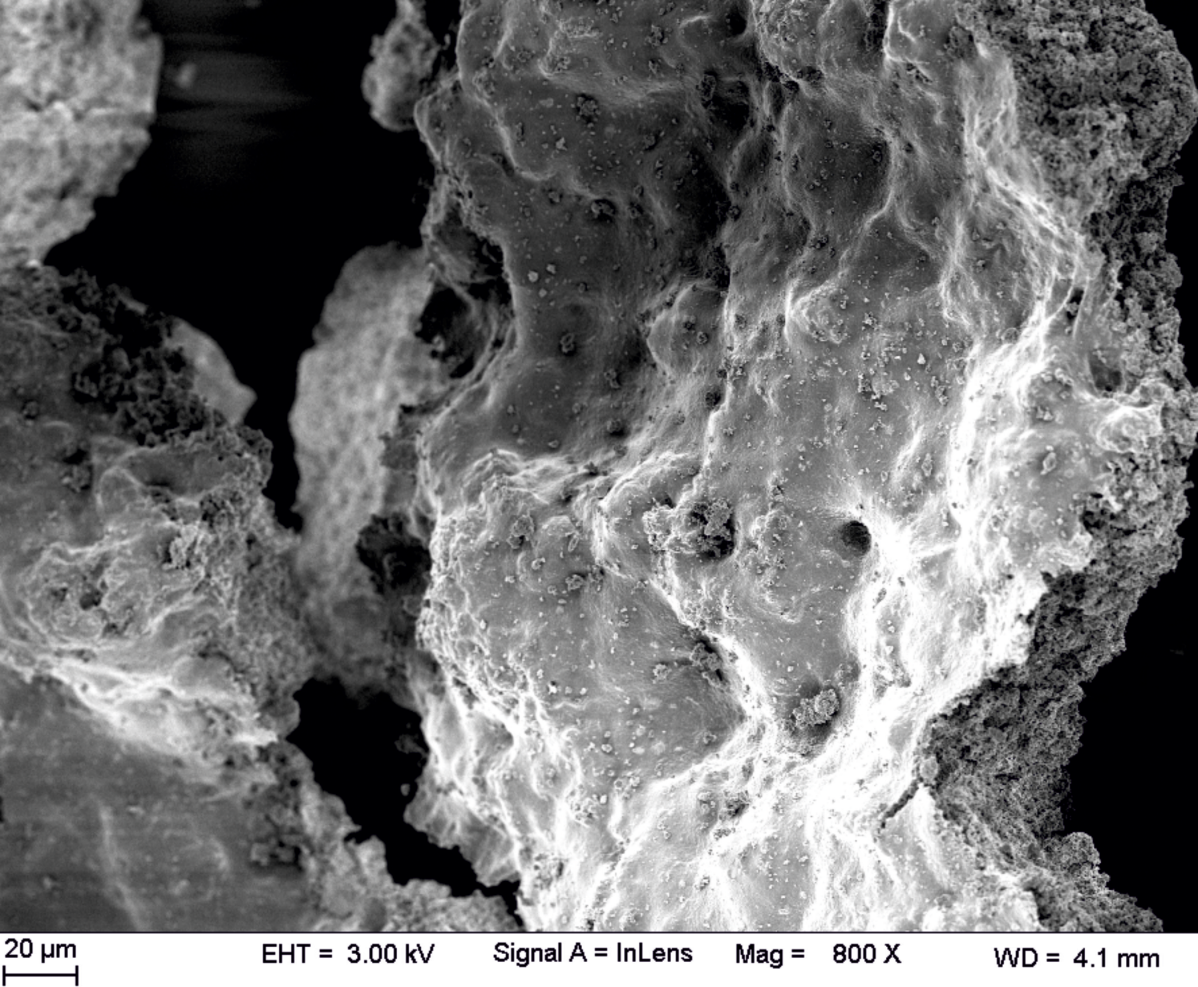
Aggregates of bacteria in the patients derived struvite crystals resembling biofilm architecture.

The FESEM images from single struvite crystals showed well-defined faces, and the porous nature of struvite was distinctly seen in all the struvite crystals (Fig 5). The pore sizes of bacterially induced struvite crystals were in the range from tens to hundreds of nanometers and the middle of the cross section in the mineral architecture displayed rougher and high porous appearance compared to control (Fig 5). Aggregates of bacteria in the struvite crystals were observed resembling a biofilm architecture (Fig 6). Naturally derived struvite stones consisted of layered pattern and exhibited void spaces with cracks inwards from the outer surfaces.

### FT-IR and XRD

The crystals showed characteristic IR spectrum with a strong peak at 1010 cm^-1^ due to the absorption of PO4^3-^ (v3 mode) antisymmetric stretch. The absorption peaks at 1469, 1435 and 1400 cm^-1^ attributed to (v4) NH_4_^+^ antisymmetric bending were also present.

**Fig 7:**
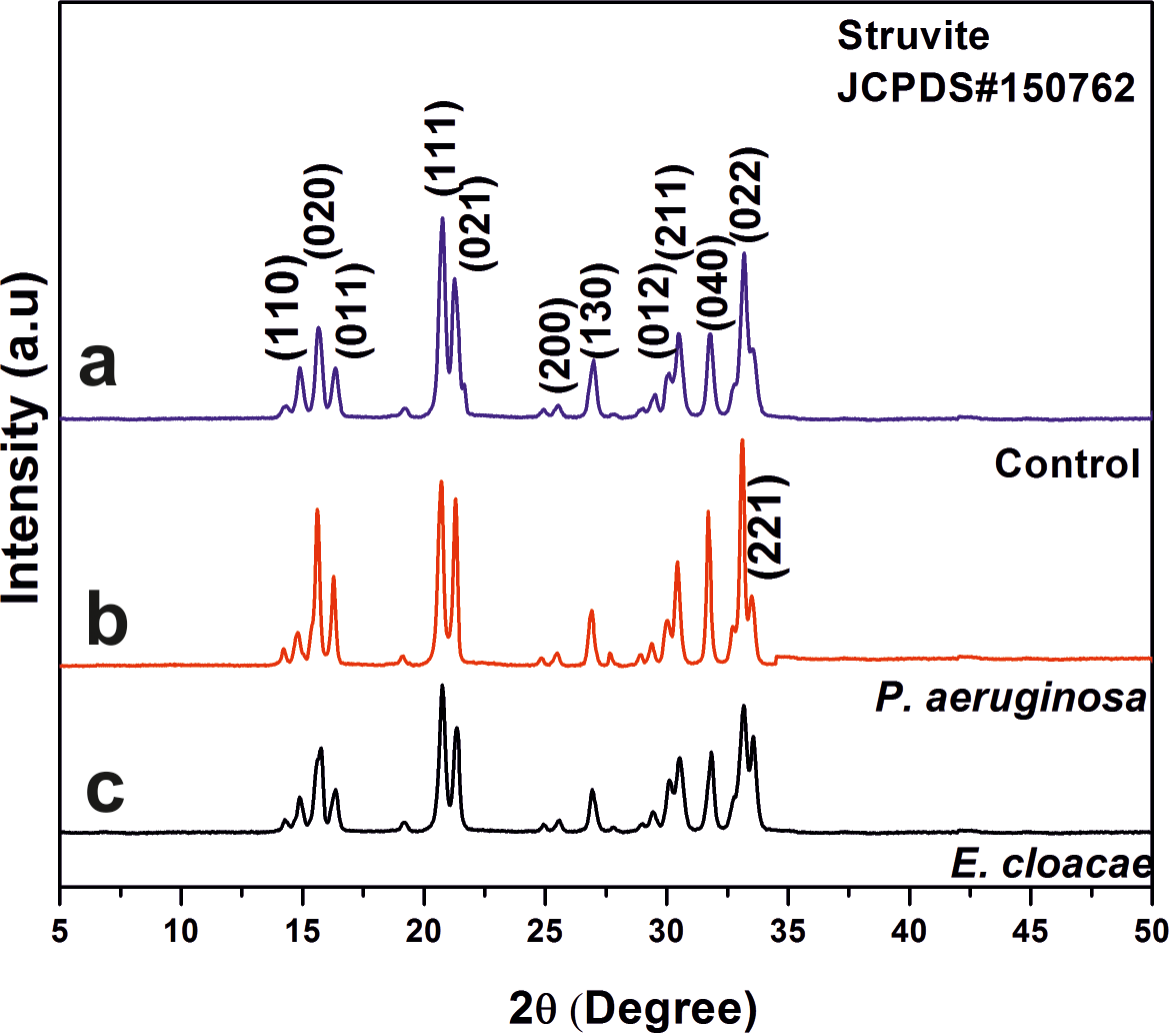
Representative XRD patterns of the of the grown struvite crystal (a) control (b) in the presence of *P*. *aeruginosa* and (c) in the presence of *E*. *cloacae*.

The XRD pattern revealed the appearance of struvite crystallizes in the orthorhombic Pmn21 space group (unit cell parameters, a = 6.955 Å, b = 11.2 Å, c = 6.142 Å). Representative XRD patterns for the crystals obtained with and without bacteria are shown in Fig 7. All the diffraction peaks were well indexed as struvite without any traces of other impurity phases. The bacteria treated samples showed higher intensity in the peak corresponding to the (221) plane as compared to the control sample.

## Discussion

The study revealed the events during the crystallization of struvite *in vitro* in the presence of two urease positive kidney stone associated bacteria. Presence of bacteria during early crystallization influenced the morphology and growth pattern of crystals. Previous study from our group reported that struvite stones can be present as pure type or along with other minerals such as calcium oxalate and calcium phosphate [21]. Struvite crystallization in the presence of urease positive strains has been investigated showing the role of bacteria in the formation of struvite *in vitro* [7, 22]. Among urease positive bacteria, *Proteus mirabilis* has been widely known for its role in biogenic struvite mineralization [22, 23]. However, the struvite mineralization using *P*. *aeruginosa* and *E*. *cloacae* are limited because majority of these strains are not urease producing.

Precise information of micro-structure during the early stages of struvite is also limited. To best of our knowledge, the present study is the first to report on struvite mineralization using these bacteria and to report the morphological transformation during the early stages crystallization using SR-μCT based imaging technique to examine the nature of bacterially induced struvite crystals. Struvite stones formed in the human kidney tend to be staghorn-like structures; and similar dendrites also appeared in bacterially induced mineralization experiments [24]. Chauhan et al. [25] suggested that *in vitro* struvite can exhibit various morphologies like pyramidal type, prismatic type, coffin shaped, needle type and dendritic type. Large dendritic structures of struvite can be formed rapidly in short time and can easily retain in the urinary tract. It can damage the epithelium of internal renal walls and enhance the crystal growth and also can easily trap bacteria [22].

Urease producing bacteria cause ammonia-induced cytotoxicity of the renal epithelium, and the increase in pH triggers the precipitation of struvite in the urinary tract [26]. The pH plays an important role during struvite crystallization. We found that struvite can form at pH between 5.5 to 9, although the best results were obtained at pH 7.2. Similar observations were reported by Clapham et al. [27]. At low pH, the struvite crystals were of coffin-like habit and at the higher pH (approximately pH 9.5), the habits of single struvite crystals remained the same, but the crystals frequently formed twins and branches.

Our time-resolved crystallization experiments suggest that in the presence or absence of bacteria, a variety morphological evolution exist and these mainly involve the formation of pin pointed-like crystals through the prismatic type to rectangular platelet type crystals, X-shape, and the flatter tabular structure with a blurred X on its surface. These characteristic X-shapes have been reported by several researchers when struvite is crystallized in the presence of urease-producing bacteria [23, 28]. *P*. *mirabilis* induced struvite crystals also show the similar morphology and habits [22]. After the formation of pin-pointed structure, it eventually leads to the formation of arrow head shaped crystals which further self-assemble and form tabular architecture with quadrangular shape. It has been suggested that, this self-assembly process is controlled by long-range dipole-dipole interactions between the assembled units [29]. Prywer et al. [22] suggested that the (001) face in bacterially induced struvite is rich in NH_4_^+^ groups, while (00Ῑ) face is rich in PO_4_^3-^ groups. Present results also suggest the intrinsic dipole-dipole interactions from the arrowhead-shaped crystals that drive them to orient towards the crystallographic directions to form quadrangular tubular architectures.

In addition to urease, bacteria can produce other metabolites and organic macromolecules that may also contribute to the promotion of crystal growth. In the absence of bacteria, relatively low amount of crystals formed, and the crystals formed in the presence of bacteria were sometimes relatively complex. Polysaccharides and proteins present in the extracellular matrices of bacteria also play a crucial role in the struvite structuration [22]. Bacterial polysaccharides containing negatively charged residues can bind the Ca^2+^ and Mg^2+^ ions to promote crystallization process [30]. Also, in the presence of bacteria, some slow-growing faces were decreased in their size, while those growing fast were increased. But, how these changes in their size affect the faces in crystal morphology is unclear.

Morphological evolution of struvite crystals as observed from SR-μCT revealed that, in the presence of bacteria, struvite crystal exhibit coffin-like morphology and these are the typical hemimorphic morphology, i.e., the two ends of a crystallographic axis are not related by symmetry. It is suggested that the biogenic X-shaped and unusual tabular struvite may represent two different growth stages [6]. We found a distinct variation in the porosity among different stages of the crystal growth in the presence and absence of bacteria. The cross-sectional architecture of the struvite in the presence of bacteria was rougher and more porous compared to control. The porosity may facilitate the adhesion of uropathogenic bacteria to form biofilms and may render bacteria resistant to antibiotic therapy or immune defense mechanisms [22].

## Conclusion

In conclusion, a series of time-course experiments with and without bacteria revealed that urease-producing bacteria such as *P*. *aeruginosa* and *E*. *cloacae* play a major role in the specific morphogenesis of struvite and these results demonstrate that the bacteria can control the nucleation, modulate crystalline phases and shape of the growing crystal. The biogenic struvite initially displayed a small arrowhead-shaped morphology subsequently evolving into large X-shaped tabular structures. Presence of bacteria can also induce transformations in crystal morphology.

## Supporting information

S1 Fig Photograph of the struvite crystals grown in gel medium in artificial urine infected with bacteria. (TIF)

S2 Fig SR-μCT images of patient derived struvite showing the layered pattern and void spaces with cracks inwards from the outer surfaces (a) projection image, (b) 2-D slice of projection.
(TIF)

S1 Table The percentage porosity of the *in vitro* struvite crystals at different stages of growth. (PDF)
