# Supplementary material for: Morphological and micro-tomographic study on evolution of struvite in synthetic urine infected with bacteria and investigation of its pathological biomineralization
Source: PLoS One. 2018 Aug 14;13(8):e0202306. doi: 10.1371/journal.pone.0202306 (PMC6091953; doi:10.1371/journal.pone.0202306)
Supplement: S1 Table — (PDF) [file pone.0202306.s003.pdf]

**Supplementary Table 1:** The percentage porosity of the *in vitro* struvite crystals at different stages of growth.

| Sl. No. | Stage | Porosity (%) | |
|---|---|---|---|
| | | **Presence of bacteria** | **Absence of bacteria** |
| 1 | $0^{th}$ | 0.00978 | 12.17147 |
| 2 | $1^{st}$ | 0.31326 | 14.56831 |
| 3 | $2^{nd}$ | 7.81515 | 11.70464 |
| 4 | $3^{rd}$ | 10.60875 | 7.97887 |
| 5 | $4^{th}$ | 11.44409 | 8.43726 |
